# How quantum computing can enhance biomarker discovery

**DOI:** 10.1016/j.patter.2025.101236

**Published:** 2025-04-29

**Authors:** Frederik F. Flöther, Daniel Blankenberg, Maria Demidik, Karl Jansen, Raga Krishnakumar, Rajiv Krishnakumar, Nouamane Laanait, Laxmi Parida, Carl Y. Saab, Filippo Utro

**Affiliations:** 1 QuantumBasel, Schorenweg 44b, Arlesheim 4144, Switzerland; 2 Center for Quantum Computing and Quantum Coherence (QC2), University of Basel, Petersplatz 1, Basel 4001, Switzerland; 3 Cleveland Clinic, 9500 Euclid Avenue, Cleveland, OH 44195, USA; 4 Deutsches Elektronen-Synchrotron DESY, Platanenallee 6, Zeuthen 15738, Germany; 5 Computation-Based Science and Technology Research Center, The Cyprus Institute, Kavafi Street 20, Nicosia 2121, Cyprus; 6 Sandia National Laboratories, 7011 East Avenue, Livermore, CA 94550, USA; 7 Elevance Health, 740 W Peachtree St. NW, Atlanta, GA 30308, USA; 8 IBM Thomas J. Watson Research Center, 1101 Kitchawan Road, Yorktown Heights, NY 10598, USA

## Abstract

Biomarkers play a central role in medicine’s gradual progress toward proactive, personalized precision diagnostics and interventions. However, finding biomarkers that provide very early indicators of a change in health status, for example, for multifactorial diseases, has been challenging. The discovery of such biomarkers stands to benefit significantly from advanced information processing and means to detect complex correlations, which quantum computing offers. In this perspective, quantum algorithms, particularly in machine learning, are mapped to key applications in biomarker discovery. The opportunities and challenges associated with the algorithms and applications are discussed. The analysis is structured according to different data types—multidimensional, time series, and erroneous data—and covers key data modalities in healthcare—electronic health records, omics, and medical images. An outlook is provided concerning open research challenges.

## The bigger picture

Precision medicine is a lofty goal, and challenges abound. A key ingredient to facilitate proactive interventions that keep an individual healthy is the detection of the earliest signals that the individual's health status is changing. Identification of such biomarkers requires advanced algorithms and analytics. Enter quantum computing. While still an emerging technology, quantum algorithms, particularly quantum machine learning, can uncover patterns that classical techniques cannot. This could enable the discovery of novel biomarkers and thus accelerate progress toward precision medicine.

## Teaser

The authors discuss how quantum computing can improve biomarker discovery. This perspective highlights the application of quantum algorithms in analyzing complex healthcare data, including electronic health records, omics, and medical images, addresses the challenges of this technology, and provides an outlook on open research challenges in this field.

## Introduction

A biomarker is a defined characteristic that is measured as an indicator of normal or pathological biological processes or of response to an exposure or intervention.^1^ The study of biomarkers arguably dates back to at least the beginning of the 20th century, with Karl Landsteiner’s discovery of the ABO blood types in 1901 (recognized with the 1930 Nobel Prize in Physiology or Medicine). Biomarkers consist of both biological molecules^2^ and other medical signs that indicate diseases,^3^^,^^4^ in contrast to symptoms as well as more general medical endpoints. Biomarkers can be based on histological, molecular, physiological, and radiographic characteristics. They may also take the form of aggregate high-dimensional features.

Common modalities of biomarkers include biomolecules, fields in electronic health records (EHRs), laboratory values, medical images, omics information, social determinants, and wearable data. Moreover, there are several application categories of biomarkers, including diagnostic, monitoring, prognostic, predictive, response, safety, and susceptibility/risk. Hence, biomarkers are context dependent and use case specific in healthcare and clinical trial settings.

For example, clinical trials in neurology and psychiatry are time-intensive, expensive, and subjective, and have lower success rates than other indications.^5^ Within neuropsychiatry, pain represents a large unmet need with respect to the lack of reliable biomarkers. Hence, quantitative and physiological biomarkers are urgently needed for a comprehensive and reliable diagnosis and assessment of pain in healthcare and clinical trials.^6^ This is illustrative of the impact that better biomarkers can have across medicine.

While the beginnings of quantum mechanics date back 100 years, quantum computing is a new form of information processing, including novel hardware and software, that leverages quantum mechanical effects such as quantum entanglement, interference, and superposition. It is the only known form of computing that can provide, at least for certain problems, exponential speedups compared with classical approaches; for other problems, there may only be polynomial speedups or none at all, however.^7^ Hundreds of use cases are meanwhile being explored across industry and academia. Quantum computing is not just a faster way of addressing problems; it also represents an entirely different way to find solutions. The benefits also go beyond speed; depending on the application and the use case, the main benefit could be accuracy,^8^ energy efficiency,^9^ or the ability to handle difficult datasets (for instance, high-dimensional or highly noisy ones).^10^^,^^11^

The focus of this review is on (gate-based) quantum computing and its potential for computational problems in biomarker discovery. Nevertheless, it is important to note that there are further quantum technologies with medical applications and biomarker relevance, in particular, quantum annealing^12^ and quantum sensing.^13^^,^^14^^,^^15^ Here, quantum computing and relevant quantum algorithms are first summarized in the section “quantum computing” before the application of quantum algorithms to biomarker discovery problems is described in the section “quantum computing for biomarkers.” Open research challenges are discussed in the section “open research challenges,” and conclusions are drawn in the section “conclusion.”

## Quantum computing

Quantum computing is a relatively nascent field of research undergoing rapid and substantial development. By leveraging the fundamental properties of quantum mechanics, namely superposition and entanglement, quantum computing presents exciting opportunities to solve problems more efficiently than classical computing can. However, the quantum computers that are available currently are prone to errors and face limitations in both the number of quantum bits (qubits) and the connectivity between them. Overcoming these constraints is essential to implementing and correctly executing any algorithm. These constraints also introduce challenges in developing algorithms to achieve a quantum advantage with available quantum devices, commonly referred to as utility-scale or near-term quantum computers. Therefore, significant efforts concentrate on utilizing near-term quantum computers, while research continues on developing fault-tolerant quantum hardware that can operate reliably even in the presence of noise.

In quantum computing, algorithms take advantage of Hilbert space. This is a high-dimensional vector space that represents the state of the qubits and is naturally suited for computing outcome probabilities (via an inner product). If the input of a quantum algorithm is classical data that is not represented by quantum states, then data embedding is required to perform operations on a quantum computer. While there are various ways to embed classical data into quantum states, the number of available qubits restricts the feature space size of the data we can tackle with near-term devices. For instance, angle encoding^16^ or instantaneous quantum polynomial encoding^17^ requires a linear scaling of the number of qubits with respect to the number of features. Alternatively, amplitude encoding provides a logarithmic scaling in the number of qubits.^16^ However, without ancillary qubits, amplitude encoding results in deep circuits.^18^ In practice, when the feature space exceeds the capacity of available quantum hardware, classical dimensionality reduction algorithms are applied as a pre-processing step in quantum pipelines. Still, with ongoing advancements in quantum computing, biomarker discovery and other quantum applications may eventually be performed without the need for significant (classical) pre-processing.

Quantum circuits are fundamental to the architecture of quantum computation, providing the framework through which quantum algorithms are designed and executed. At a high level, a quantum circuit is a sequence of quantum gates applied to qubits. Each gate performs a specific operation on one or more qubits. Fault-tolerant quantum algorithms typically require running deep quantum circuits on tens of thousands to millions of qubits, making them unsuitable for near-term devices. However, variational quantum algorithms (VQAs)^19^ utilize shallower circuits and can produce meaningful results. This characteristic has made VQAs highly popular for near-term quantum computing applications. VQAs are hybrid quantum-classical algorithms that involve an iterative loop between quantum and classical hardware. First, quantum circuits that have parametrized gates, called the ansätze or parametrized quantum circuits (PQCs), are employed to obtain samples from a quantum computer. Second, a classical computer optimizes the parameters of the quantum circuit with respect to the loss function of the problem. Most quantum machine learning (QML) models belong to the class of VQAs and can be evaluated on quantum computers. Alternatively, quantum computers could be leveraged as sub-routines within classical algorithms. One example is the thermal state preparation during Boltzmann machine training. However, the performance of VQAs is highly sensitive to hardware noise, necessitating the use of error mitigation techniques to partially counteract noise-induced errors.^20^

The execution of quantum circuits is performed on quantum hardware through quantum processing units (QPUs), in analogy to central and graphics processing units. Another element is the quantum random access memory (QRAM), which is a quantum memory that stores a quantum state for later retrieval. Unlike QPUs, which are readily available via cloud computing platforms, hardware incorporating QRAM devices has not yet been demonstrated.

Quantum computing is naturally suited for quantum data (which describes quantum states). However, it also holds great promise in solving problems using classical data. In particular, there has been much progress in the last few years in understanding how quantum computers can solve computational problems that can be formulated as cryptography, ML, optimization, or simulation (of nature) problems.^21^ In this paper, we focus on analyzing classical data with the help of quantum algorithms, including multidimensional, time series, and erroneous data, which is discussed in detail in the section “quantum computing for biomarkers.”

### QML

QML integrates principles from quantum computing and ML, promising potential advances in learning from data. A recent benchmarking study^22^ provides a comprehensive evaluation of popular QML models on binary classification problems. The study examines QML architectures compatible with near-term quantum devices such as quantum neural networks (QNNs), including quantum convolutional neural networks and quantum kernel methods (QKMs).

The majority of QNNs are based on PQCs. The parameters of PQCs are optimized following the VQA paradigm. A typical QNN begins with data embedding, followed by a series of gates. One of the most general QNN models for classification is the variational quantum classifier (VQC), which is essentially a PQC. Another example of a VQC is the circuit-centric quantum classifier,^23^ one of the earliest proposed generic QNNs. The data re-uploading classifier is a notable QNN architecture that was shown to have universal approximation properties.^24^ The data re-uploading classifier features a processing layer composed of data encoding and unitary operations with trainable parameters. This layer is repeated a number of times, each layer having independent parameters.

Recently, significant attention has been given to leveraging quantum computers for evaluating kernel functions in kernel methods. Quantum kernels, based on feature maps (data encodings), could be used with classical maximum margin classifiers. To evaluate a quantum kernel function, a circuit is designed by first applying a feature map and then its Hermitian conjugate. A prominent example of data-dependent quantum advantage in QML models is demonstrated through QKMs. Huang et al.^25^ established a relation between the potential advantage in predictive accuracy of a model for a given dataset and a quantum kernel. In addition, a projected quantum kernel classifier was introduced.

Although the predictive advantage of QML models for practically relevant problems remains an open question and necessitates data embeddings that are difficult to simulate classically, quantum models are expected to show better generalization than classical models, leveraging fewer data points.^26^ This is particularly intriguing in fields where constructing sufficiently large datasets to ensure satisfactory performance for ML models is challenging, as is the case in many healthcare settings.

The findings by Bowles et al.^22^ highlight the significance of experimental design for the performance of quantum models. Problem-agnostic implementations of QML models are unlikely to outperform their classical counterparts, highlighting the need for tailored approaches. Specifically, the embedding of classical data into Hilbert space and subsequent operations should be tailored to an application and available hardware. Additionally, for QML models based on VQAs, the barren plateau (BP) phenomenon should be addressed. A model experiences a BP when its loss landscape flattens, and the variance of parameters’ gradients decays exponentially as the system size increases, making gradient-based optimization challenging. Avoiding BPs in QML models commonly necessitates reducing the expressivity of the circuits, which also tends to make them more classically simulable (decreasing the potential of significant quantum advantages).^27^^,^^28^ These insights underscore the importance of use case-specific algorithm design for the potential benefits of quantum computing.

Despite the challenges in algorithm design, a variety of (machine) learning tasks are being explored with quantum algorithms. An overview of learning tasks, use case examples, and corresponding quantum algorithms (many of the algorithms may also be relevant to other learning tasks and use cases) is given in Table 1 and is further discussed below.

**Table 1:** Overview of machine learning and related tasks and examples of use cases and quantum algorithms

Learning task	Use cases	Quantum algorithms
Dimensionality reduction	covariates for biomarker trait regression models	QPCA,^29^^,^^30^ QLDA,^31^ QSFA,^32^ QIsomap^33^
Classification	early disease stage prediction	QNNs,^23^^,^^24^^,^^34^^,^^35^ QKMs^17^^,^^25^
Regression	disease risk prediction	quantum linear regression^36^^,^^37^^,^^38^^,^^39^
Clustering	multi-omics data analysis	QK-means,^40^^,^^41^ quantum spectral clustering^42^
Generative learning	synthetic medical images	QNNs,^43^ QBMs,^44^^,^^45^ QGANs^46^
Time series forecasting	longitudinal patient studies, drug-resistance mechanisms	QRC^47^
Natural language processing	clinical notes processing	QLSTM^48^
Optimization	treatment optimization	QAOA,^49^ QPSO,^50^ VQE^51^

Consider the example of dimensionality reduction, a key technique used to compress large and complex datasets while preserving key information. As quantum computing rapidly develops, quantum algorithms for reducing the feature space size are evolving alongside it. For instance, many classical dimensionality reduction algorithms have a quantum adaptation, such as quantum principal-component analysis (QPCA),^29^ quantum linear discriminant analysis (QLDA),^31^ quantum slow feature analysis (QSFA),^32^ and quantum isomap (QIsomap).^33^ A QPCA speedup on quantum data is highlighted in the work of Gordon et al.^30^ However, the majority of the quantum algorithms for dimensionality reduction still require further algorithmic advancements and fault-tolerant hardware to achieve practical relevance.

Many other learning tasks may benefit from quantum computing. This includes supervised learning, regression analysis being one example, for instance via quantum linear regression.^36^^,^^37^^,^^38^^,^^39^ Likewise, unsupervised learning may benefit from approaches such as quantum k-means (QK-means) clustering^40^^,^^41^ and quantum spectral clustering.^42^ Given the recent rise of generative artificial intelligence (AI) techniques, quantum generative models are receiving renewed interest, including QNNs,^43^ quantum Boltzmann machines (QBMs),^44^^,^^45^ and quantum generative adversarial networks (QGANs).^46^ As a final example, natural language processing is an emerging area that has recently progressed through the emergence of quantum natural language processing (QNLP).^48^^,^^52^

## Quantum computing for biomarkers

Quantum computing is not a silver bullet; it does not enable improvements for every single computational task and thus not every problem in biomarker research. Therefore, it is important to categorize and narrow down which (important) problems in biomarker research are particularly suited in regard to the application of quantum algorithms. Related work has been conducted for the biological sciences,^53^ cell-centric therapeutics,^54^ clinical trials,^55^ digital health,^56^ and health and medicine.^57^

**Figure 1:**
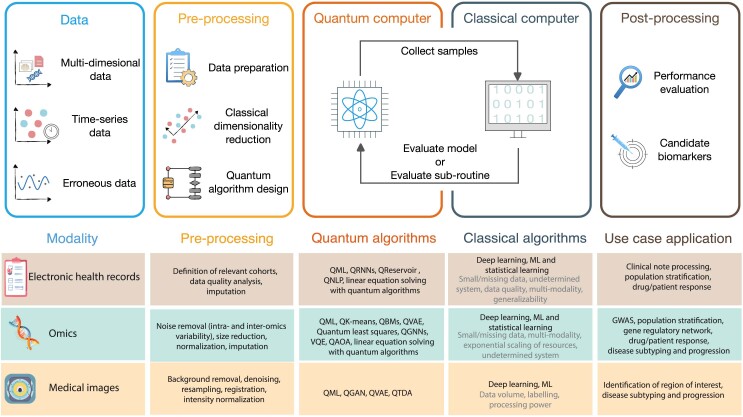
Flow of data through a hybrid quantum-classical computational pipeline for different types of data and different healthcare data modalities The top part of the figure focuses on the categories multidimensional, time series, and erroneous data. The bottom part covers the modalities EHRs, omics, and medical images. Examples are given for pre-processing steps, quantum algorithms, classical algorithms and their limitations (in gray), and medical use case applications.

In this section, we organize areas of opportunity in applying quantum computing to biomarker discovery along the lines of data types while discussing multiple healthcare data modalities for each data type. We chose this categorization instead of one explicitly centered on healthcare data for the following reasons. Quantum algorithm development advances at a fast pace, and research in that field often makes explicit assumptions about data types as well as quantum computer accessibility. As such, by focusing on connecting data types to healthcare modalities, it is our hope that insights provided by this perspective retain relevance in the face of future developments in quantum algorithms. Moreover, this allows insights from the given perspective to have stronger cross-disciplinary relevance. Specifically, in this paper, we organize around multidimensional data, time series data, and erroneous data. For each of the above data types, their occurrence in and relevance to different healthcare data modalities are analyzed in the context of the applicability of quantum algorithms. Figure 1 illustrates the flow of data through a hybrid quantum-classical computational pipeline for different types of data, healthcare data modalities, classical approaches and limitations, and pertinent quantum algorithms for selected use cases.

After preparing the data for downstream analysis, classical dimensionality reduction techniques may be applied due to the limited number of available qubits of near-term quantum devices. These may differ between EHRs,^58^ omics data,^59^ and medical images.

Clearly, the flow illustrates that quantum computing algorithms are only inserted at very specific points of the computational pipeline; most computational steps remain on classical systems. Optimizing the quantum part requires, for example, adapting the algorithm to both the specifics of the device and the characteristics of the data. The classical data should be embedded into a Hilbert space via data encoding techniques.^60^ Depending on the nature of the data and the specific problem at hand, it may be necessary to develop data encoding methods tailored to the application. Additionally, the quantum algorithm should account for the quantum device’s connectivity and its limitation. For instance, error mitigation and suppression protocols^20^ should be considered prior to running the algorithm to address potential hardware-induced errors.

### Multidimensional data

Recent technological developments have produced an abundance of data in the domains of healthcare and life sciences.^61^ These are often correlated in intricate ways, which may necessitate a (quantum) network model approach^62^ and ML techniques such as (quantum) graph neural networks.^35^^,^^63^

Furthermore, multidimensionality of data can occur due to different modalities and time changes for a set of samples and features. Medical imaging, next-generation sequencing, and other high-throughput instruments produce streams of multimodal data that are referred to as “big data.” In the context of biomarker discovery, these massive data volumes acquire orders of magnitude more quantity and complexity when utilized in large-cohort analyses such as genome-wide association studies (GWASs). A key problem, however, arises in the dimensionality of the data. Even big data suffers from having too many features compared with the number of samples, which significantly complicates data science.^64^ While there are solutions for increasing the number of samples, these are often still limited for biological datasets. Methods for dimensionality reduction and feature selection therefore become extremely important in handling big data so that the dimensionality can be returned to where there are many more samples than features. This then blurs the lines between big data and “small data,” which can be very limited in either the number of samples or the number of features. Small data with few samples is often encountered in population studies of rare diseases and conditions. Even though the underlying modality (e.g., whole-genome sequencing data) is more aptly classified as big data, the intrinsically limited number of samples (e.g., in the case of individuals diagnosed with a rare condition) constrains a biomarker discovery investigation to small data.

In all regimes of data (big, small, and the entire spectrum in between), quantum computing presents us with new opportunities (and a new set of challenges) to address persistent limitations of classical computational approaches. One notable example is overcoming the data and scale inefficiencies of modern classical ML. In the past decade, ML has made incredible advances powered by massively scaling training data, computing budgets, and the sizes of deep neural networks. Recent theoretical results have shown, however, that the efficacy of deep neural networks is tied to the number of samples accessible during the training phase and the effective dimension of the dataset. To illustrate this fundamental relationship, training current neural networks to achieve a robust generalization error on a dataset roughly the size and effective dimension of ImageNet would require a model with around 10–100 billion parameters,^65^ rivaling the size of some of the biggest large language models trained to date. Empirical studies by various research groups have confirmed this unfavorable scaling of model size, data size, and computing budget for other data modalities such as natural language.^66^^,^^67^ Recent research in quantum computing points to the possibility of a new class of quantum algorithms that are both data and model size efficient and able to generalize to new data with few data samples.^34^

While there is evidence for more data-efficient QML algorithms, the loading of classical data into quantum computers represents a new set of challenges that exists in both current and near-term quantum hardware. How quantum computers can access data is of central importance in the field of QML. In fact, some early QML algorithms made assumptions around access to classical data that have been shown to lead to computational advantages over their classical counterparts. However, once their data access assumptions were closely inspected and loosened, it was found that many of those computational speedups no longer hold.^68^ Going deeper into the topic of quantum access to data is beyond the scope of this perspective; suffice it to say that for classical big data, data loading may erase computational quantum advantages. Different research efforts are under way, such as QRAMs^69^ on the hardware side and coresets^70^^,^^71^ on the algorithmic side, to address this challenge. For the time being, however, small data is a more natural fit for existing and planned quantum computers.

#### EHRs

The increasing adoption of EHRs has revolutionized biomedical research. EHRs provide rapid data availability, eliminating the need for extensive recruitment or data collection. Additionally, they support longitudinal tracking of patient health, facilitating disease progression analysis. Their ability to capture data from diverse patient populations also enhances the detection of subtle health trends.

For the scope of this paper, EHRs are assumed to contain (typically structured) information such as diagnoses, immunization, laboratory measurements, medications, procedures, and vital sign data as well as unstructured doctor notes. They also increasingly integrate social determinants of health (SDOH) and wearable data. Medical images and omics, due to the explosive growth in their collection and relevance, are discussed separately in this paper.

SDOH, encompassing economic stability, education, healthcare access, neighborhood environment, and social context, significantly impact health outcomes. While some SDOH data (e.g., age, location) can be extracted from EHRs, other data (e.g., social and behavioral determinants) is often missing. Since SDOH are strongly correlated with morbidity and mortality, integrating them with EHRs is crucial. However, the complex interplay between health and social factors presents significant analytical challenges.^72^

The application of deep learning, ML, and AI to EHRs has yielded valuable medical insights, including accurate prediction of future health conditions.^73^^,^^74^ AI-based approaches^75^^,^^76^ broaden the understanding of biomarkers, particularly when EHRs are fused with other data sources.^77^ The construction of such multimodal datasets may itself also benefit from AI techniques.^78^^,^^79^ Therefore, the increasing integration of EHRs in biomedical research suggests that QML holds immense potential for advancing biomarker discovery. In particular, quantum computing could enhance the analysis of EHRs in small-cohort studies, such as clinical trials or rare disease research, by improving predictive accuracy from small datasets.

Furthermore, QNLP shows promise in representing linguistic information more effectively than classical methods.^48^^,^^52^ For example, clinical notes may show that an epilepsy patient has been seizure-free,^76^ which would otherwise likely have to be indirectly inferred from the absence of medical codes. Therefore, QNLP methods may be applied in analyzing free-text doctor’s notes, which offer valuable insights.^80^

#### Omics

Handling sparse omics data is often a limitation for classic ML and statistical techniques in biomarker discovery. Quantum computing may actually provide a solution to some of these challenges.

Genomic, proteomic, and other omics data have revolutionized our understanding of complex diseases. Spurred by technological advances (including next-generation sequencing), omics often includes high-throughput empirical studies, producing raw data that necessitates multistep computational processing and sophisticated analysis before being amenable to interpretation. In the last decade, omics have become widely used in biomedical research to study disease mechanisms, identification of biomarkers for therapeutic development, and diagnostics in the clinical setting.^81^^,^^82^ Although such large datasets can now be generated, biological data suffers from what is known as the “curse of dimensionality,”^83^ which makes it challenging to perform traditional ML, due to overfitting, and to properly train the model. Mitigation of this is aided by state-of-the-art methods in single-cell sequencing and spatial transcriptomics, which begin to address the issue of dimensionality by interrogating individual cells rather than populations. While the number of patients will not increase with single-cell analysis, having information from individual cells allows for more detailed biomarker identification strategies, especially in diseases with heterogeneous manifestations such as cancer. However, even with single-cell analysis, limited data and too many features continue to be problems, and addressing these problems requires techniques such as dimensionality reduction, feature selection, and clustering.^84^

An example is biomarker discovery for the stratification of patients into different groups based on their susceptibility to a particular disease or their response to a specific drug. Often, such studies are limited in the number of available samples, but owing to the large number of features, the search space can be vast. Indeed, collecting omics data from patients is challenging for many reasons, most notably the invasive nature of the medical procedures, their associated costs, and a lack of infrastructure. This is an area where quantum computing has already had demonstrable impact and will continue to be increasingly impactful as data availability increases and the hardware and software evolve.^85^^,^^86^

Furthermore, with genome sequencing becoming increasingly democratized, methods such as GWAS and quantitative trait loci (QTLs) mapping are often used to identify genetic biomarkers of disease states or phenotypic variation in populations.^81^^,^^87^ However, for approaches such as GWAS and QTL mapping to reach statistical significance, let alone train predictive models, huge amounts of complex data would be required, and this is where quantum computing can provide potential alternatives to classical algorithms.^88^ In addition, GWAS studies often require methods such as PCA to identify covariates for biomarker trait regression models.^89^ In cases where the covariance matrix of interest is of a low rank, QPCA^29^ and variants thereof^30^^,^^90^ can prove far more efficient than their existing classical counterparts, albeit issues of data loading and quantum hardware fault tolerance must be overcome first.

The interpretation of genetic variants in terms of biomarkers is typically limited by inherent correlations between causal and non-causal variants,^91^^,^^92^^,^^93^ requiring formal causal inference approaches. The latter are notorious for their prohibitive computational cost,^94^ except in cases with restrictive assumptions about the underlying causal structure. Quantum causal inference is an active area of research that could provide more powerful causal discovery algorithms than are currently available classically. So far, quantum speedups in causal inference have been obtained only for datasets whose statistics cannot be described entirely with classical means due to the presence of quantum correlations.^95^

Another prevalent approach to biomarker discovery with omics data is through studies of how the transcription of one gene affects the transcription of its neighboring genes, whether it be through statistical approaches such as expression QTLs or gaining predictive power from gene expression patterns through ML.^96^^,^^97^ An efficient way to rapidly understand and analyze complex regulatory relationships between genes is through gene regulatory networks (GRNs).^98^^,^^99^ GRNs are computationally efficient since they only include pairwise interactions. Recently, a PQC model for a quantum single-cell GRN was proposed to try to allow for higher-order correlations and preserving the computational efficiency of GRNs.^100^ Authors have analyzed single-cell RNA sequencing data from lymphoblastoid cell lines originating from two data sources.^101^^,^^102^ Mixed results were found with the quantum models, with some correlations in accordance with previous findings, other correlations in contradiction to previous findings, and even new correlations that were not present in the baseline model. This work is an example of how novel quantum computational models and algorithms, even when co-designed with omics problems, can lead to new insights and help bypass the data-uploading challenges described earlier.

Moreover, an additional avenue to understand the co-expression of genes is to implement methods to cluster gene-expression datasets. While there are many techniques for clustering gene expression data,^103^ more traditional methods often make assumptions about the data or make *a priori* decisions about parameters that are not necessarily sound.^104^ To circumvent this, more complex algorithms such as genetic algorithms or particle swarm optimizers (PSOs) have more recently been explored for genomic clustering, with PSOs showing promising results with respect to validated accuracies compared with other methods.^105^ However, the disadvantage of this algorithm is that it does not guarantee a convergence to a global minimum^106^ and is prone to being trapped in local optima.^107^ Classical techniques to overcome this drawback have been discovered, but their implementation can be computationally expensive.^108^ A potential solution to mitigate this issue is to use the quantum-behaved particle swarm optimization algorithm^106^^,^^109^ and its modified versions.^110^^,^^111^^,^^112^ The idea is to suppose that each point-particle is now a spinless one with quantum behavior that follows a corresponding Schrödinger equation, which can then be used to perform the particles’ position and velocity updates at each iteration. Although the references cited have implemented these quantum-behaved PSO algorithms on classical computers, to our current knowledge, they have not been discussed in the context of being implemented on a quantum computer. It appears possible that furthering the understanding of implementing quantum-behaved PSOs on quantum computers, in addition to exploring other quantum optimization techniques (not necessarily focused on clustering) such as the quantum approximate optimization algorithm (QAOA) and the variational quantum eigensolver (VQE), could lead to quantum advantages.

#### Medical images

Medical images are a widely used approach for assessing injury, disease, and health. By using techniques such as X-ray, magnetic resonance imaging (MRI), computed tomography (CT), positron emission tomography (PET), and ultrasound, imaging enables physicians and researchers to visualize internal structures and functions of the human body. Qualitative medical imaging involves the visual interpretation of images by trained healthcare professionals to identify and characterize abnormalities, lesions, or anatomical structures by focusing on subjective observations such as size, shape, texture, and density to make diagnostic assessments. This raises issues when it comes to decision-making with images.

Even when looking for known features in medical images, professionals can miss details or find specific images that are difficult to interpret. While algorithms exist for dimensionality reduction and feature selection of images, the amount and diversity of data that need to be processed can often be a computational challenge and lead to insights being missed. This is where quantum computing could play a significant role. Quantum algorithms,^113^ particularly QML,^114^ have shown promise in this application space.

Consider the case where a radiologist may identify the presence of a tumor in an MRI scan based on its appearance and contrast with surrounding tissues. Quantitative medical imaging involves the extraction of numerical or quantitative data from medical images using computer-based algorithms and software tools. Here, subjective measurements of various parameters such as blood flow, density, metabolic activity, and volume are obtained, which may allow for more precise and standardized assessments of disease severity, progression, and treatment response. An oncologist may use quantitative imaging techniques that can measure tumor size, shape, and growth rate to assess treatment efficacy and predict patient outcomes. To be able to do this, significant computing power and advanced computational techniques are needed; traditional approaches are often not sufficient.

Another issue that arises is when specific features are not known, and exploratory analysis is required for diagnosis, which exacerbates the computing problem. For example, although approximately 1.2% of the US population has active epilepsy,^115^ only half of these cases have a determined cause. In cases where identification is possible through known markers, MRI is commonly used to detect structural brain abnormalities, such as hippocampal sclerosis, cortical dysplasia, and brain tumors, which may be the underlying cause of epilepsy. In cases where structural abnormalities are not observed, PET can be used to detect areas of hypometabolism that may correspond to epileptogenic zones (EZs). Single-photon emission CT can be used to visualize blood flow changes during a seizure and between seizures, where the comparison of these images can aid in localizing the EZ. Hybrid imaging combines several different imaging techniques, leveraging both structural and metabolic information to improve localization. Extensive analysis is still required, however, to identify more markers that encompass a wider spectrum of epilepsy patients and provide more granular detail into the type and severity of the disease, which in turn requires massive amounts of data collection and processing.

Reconstructing images from lower-dimensional and lower-resolution data is another area of potential quantum enhancement.^116^ For example, quantum techniques for volume-rendering medical imaging data more efficiently have been proposed.^117^ The challenges in medical imaging go beyond image processing. Medical imaging equipment often comprises devices that require specialized environments and maintenance. For instance, MRI machines need high-powered magnets and must be kept at a temperature of a few degrees kelvin. Lower-powered magnets that do not require such extreme environments because they benefit from quantum algorithms that place lower demands on image resolution would be a boon for accessibility and equity.

### Time series data

The models used in predicting future values of a time series given a set of past values are fundamentally different from predicting labels or a quantity given a set of features. This is because the former is an extrapolation whereas (in most cases) the latter is an interpolation. Thus, common models (ML or otherwise) used in classification or regression problems usually do not perform well for time series prediction and forecasting, and time series data require other approaches.

For many biomedical applications, analysis of time series data, also known as longitudinal data, presents a range of challenges for classical techniques, including ML models. Some of these challenges are algorithm selection, explainability, heterogeneous data, inconsistent labeling and annotations, missing data, and unbalanced data.^118^ In addition, the many correlated variables typically found in biomarker data make it difficult to elucidate causal pathways.^119^ Quantum computing techniques, particularly QML algorithms such as quantum reservoir methods,^120^ are actively researched to achieve enhanced time series processing.^121^

There already exists a variety of techniques to tackle time series problems. For many examples, one can often obtain reasonable predictions by using relatively simple models such as autoregressive moving average models and their many variants.^122^ For time series data with additional challenges, such as the ones mentioned at the start of this section, the current state-of-the-art is arguably the long short-term memory (LSTM) ML model,^123^ which is able to capture some properties, such as seasonality and highly correlated variables, much better than the more traditional models. However, a recent QML technique that has also started to gain attention is quantum reservoir computing (QRC).^124^ Reservoir computing is a predecessor to recurrent neural networks (RNNs), where the hidden weights can be treated as a reservoir that projects the data into a higher-dimensional subspace, which can then be fed into a simple linear model; quantum analogs exist.^125^ The reservoir (similar to the RNN) receives the time series data sequentially, and hence the dynamics of the reservoir are dependent on the previous time step. This reservoir can have many different forms, ranging from classical architectures like the echo state network^126^ to physical implementations.^127^^,^^128^ The advantage of reservoir computing versus RNNs is that since the majority of the randomly initialized parameters of this reservoir is fixed from the start, the computational cost of training them is much lower. However, this randomness typically requires reservoirs to have many more parameters to achieve comparable accuracy. Still, the use of a quantum reservoir (e.g., by generating a random quantum circuit) can decrease the number of parameters required for the model to perform well.

#### EHRs

Time series analysis is particularly relevant to EHRs, given the longitudinal nature of many of the components (e.g., laboratory values). To move closer to proactive medicine, one needs to be able to accurately predict the next points in a time series to achieve early diagnostics and interventions. EHRs suffer from many of the data challenges described earlier. For instance, many of the patients go in and out of healthcare systems, and as a result, approaches such as hidden Markov models (HMMs) are applied.^129^ Although these models are good candidates to handle noisy and highly variable signals, they tend to fall short for several reasons, including the high memory requirements to implement these models at scale and attempts to take into account all the important variables. Using QRC or even a quantum version of HMMs could relieve this bottleneck around high-memory requirements if the data can be encoded in an efficient way.

Furthermore, wearables represent another modality that is growing rapidly and starting to be integrated with EHRs.^130^ These include chemical sensors that collect signals with electrochemical and optical methods,^131^ as well as fitness trackers and other devices that monitor digital biomarkers.^132^ They may also include quantum sensors that exploit quantum mechanical properties to achieve superior sensitivities, such as nitrogen-vacancy-center-based magnetometry at the level of single neurons.^133^ By their nature, these wearables typically generate time series data and could thus benefit from such quantum (ML) methods.

#### Omics

Omics time series data are particularly valuable for biomarker discovery, as they allow the observation of temporal patterns and gene-gene/protein-protein interactions. Examples includes disease progression/sub-typing^134^^,^^135^ and patient response to treatments.^136^^,^^137^^,^^138^^,^^139^

Non-invasive approaches to use liquid biopsies allow one to capture a mixture of the omics profile of cancer cells in a patient via cell-free DNA (cfDNA). Recently, studies have demonstrated the application of cfDNA to identify drug resistance mechanisms.^140^ Moreover, in the last few years several studies have shown that cfDNA fragmentomics’ characteristics differ in normal and diseased individuals without the need to distinguish the source of the cfDNA fragments, which makes it a promising novel biomarker in particular for early cancer detection.^141^^,^^142^ Indeed, ML algorithms have been applied to such tasks with promising results to identify late-stage cancer^141^ but still fail to detect early signs in the development of the disease. Another recent application of omics time series is in the case of epilepsy post-surgical seizure recurrence,^134^ where omics data were used to subtype patients based on their treatment responses.

In general, classical ML often cannot be easily applied to omics-based time series data due to issues such as small sample numbers or missing data. Therefore, data imputation is required, and current imputation techniques often do not incorporate time dependence, thereby leaving out critical information during imputation. Recent work has demonstrated that graph networks that model topology across samples can be hugely beneficial in such cases.^143^ An example is the use of dynamic Bayesian networks for analysis of time series microbiome data,^144^ which has been demonstrated to be a critical biomarker for many disease states.^145^^,^^146^ Still, these topological methods quickly become computationally infeasible with increasingly high-dimensional and complicated datasets, which is where QML and quantum topological data analysis (QTDA)^147^ approaches may be able to help, providing an orthogonal point of view and insights. Indeed, they could be used to differentiate with high accuracy between healthy individuals and cancer patients in early stages of the disease, leading to more targeted and effective treatments.

#### Medical images

Medical image time series data occur across a continuum of modalities, ranging from a set of snapshots separated in time to movie-like videos. Acquiring image time series data for biomarkers involves capturing medical images over time to monitor changes in biological or physiological states. Examples of time-lapse series include (1) MRI or CT scans taken over weeks or months, showing the size and shape of a tumor changing in response to treatment and acting as a biomarker for cancer therapy efficacy; (2) retinal imaging that tracks the progression of diabetic retinopathy via blood vessel leakage, aneurysms, and hemorrhages; and (3) PET scans of the brain, showing changes in glucose metabolism or amyloid plaque accumulation for neurodegenerative diseases such as Alzheimer disease. Examples of movie-like imaging data include four-dimensional ultrasound scans (the fourth dimension being time) for fetal development and function and functional MRI to observe changes in brain activity in response to stimuli.

QRC techniques are relevant techniques for application to these problems. However, the quantum computers would have to be larger than the current generations or the initial image data would have to be downscaled with classical pre-processing techniques, such as passing the image through a residual network^148^ before sending it through the QRC model. Further investigation is required to understand whether the loss of information during the classical pre-processing would negate the potential advantages of using QRC and related quantum methods instead of classical techniques.

### Erroneous data

Noise and errors often appear in the context of quantum computing due to the fragility of the quantum systems and their inherent tendency to decohere. A variety of approaches is being researched to handle or even leverage such noise^10^^,^^149^^,^^150^^,^^151^^,^^152^ and progress toward quantum error correction,^153^ arguably the holy grail of quantum computing. The focus of this discussion, however, is on handling noisy datasets where the labels are incorrect or missing, which represents a major challenge for classical algorithms.^154^^,^^155^^,^^156^ Such misannotations are also of concern when data are synthetically generated,^157^ an area that is also being explored with QGANs and related methods.^158^

#### EHRs

EHR data have been notoriously difficult to analyze due to factors such as errors, lack of interoperability, record gaps, and unusually large or small data sizes. While leveraging such data may also allow better generalization behavior through classical models,^73^ QML has shown promise in dealing with such difficult datasets even more effectively. For example, the usual presence of quantum noise may actually lead to a greater degree of global robustness and thus fairer models.^159^ Moreover, quantum transfer learning may be beneficial when the (real-world) dataset of interest is particularly low or high dimensional.^160^ Quantum support vector machines have been applied to EHRs and have, for small datasets, been shown to be competitive with classical approaches in classifying ischemic heart disease^161^ and predicting the persistence of rheumatoid arthritis patients.^162^ Small datasets, for instance, for rare-disease or other very restricted cohorts, may in fact lead to some of the earliest quantum advantages, given that quantum generative and other QML models are particularly suitable for such a setting.^26^^,^^163^ Finally, the process of imputation itself may be enhanced, leveraging quantum determinantal point processes.^164^

#### Omics

Erroneous omics data, often caused by sequencing errors, incomplete data, or misinterpretations, can significantly hinder accurate analysis. These inaccuracies can lead to misdiagnosis, ineffective treatments and loss of trust in omics data. Quantum algorithms can enhance pattern matching/discovery and error correction in omics sequences, making it possible to identify and correct errors.^88^ A possible approach could be the use of QTDA, which helps characterize the shape and structure of the data in a high-dimensional space (involving Betti number and harmonic representations), making it more effective than traditional methods in regard to identifying and correcting errors in complex omics data. Such techniques not only enhance the quality of the omics data but also accelerate the discovery of biomarkers.

#### Medical images

Erroneous data in medical imaging can lead to misdiagnoses, poor treatment outcomes, and overall inefficiencies in healthcare. Examples include noise, artifacts, and image reconstruction issues. Medical images can often display distortions caused by equipment limitations, environmental factors, and patient movements.

Quantum algorithms that have been designed for noise reduction and denoising^165^^,^^166^ may be able to more efficiently and effectively process noisy imaging data and distinguish between meaningful information and noise. Due to the large amount of data generated by medical image technology, the compression, storage, and reconstruction of such data remain significant challenges. In many cases, raw imaging data (such as k-space data from MRI) are deleted shortly after acquisition. Quantum-assisted data compression, coupled with QML approaches, may be able to more effectively compress large medical images without losing critical information.^167^^,^^168^ Finally, as image reconstruction is a complex task that is further complicated by erroneous data, reconstruction algorithms must be sufficiently robust to be able to handle noise and mistakes.^169^ Quantum algorithms and QML techniques may be more effective at detecting patterns in medical images, leading to increased capabilities in indicating and resolving such erroneous imaging data.

## Open research challenges

**Figure 2:**
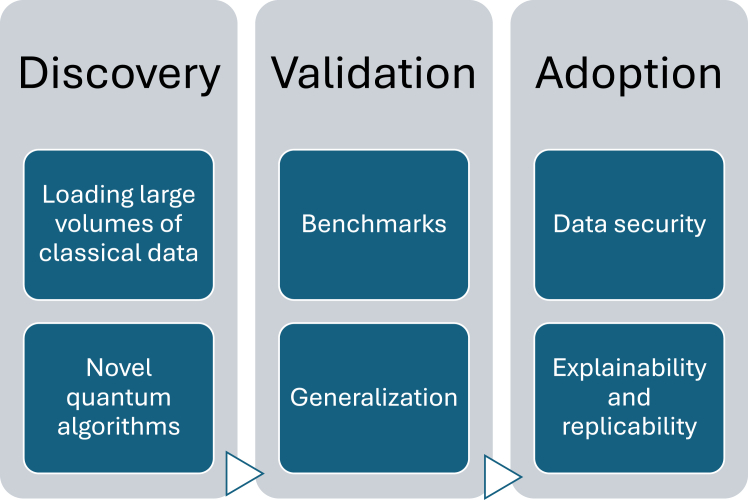
Overview of open research challenges in the application of quantum computing in biomarker discovery, validation, and adoption

Quantum computers are swiftly moving from lab to industry. A broad range of improvements are nevertheless still necessary on the road to commercialization and widespread usage. Many of the open problems are cross-industry; better quantum hardware, algorithms, and software will enable more and more use cases. In this section, some of the challenges that are particularly relevant to biomarker discovery, validation, and adoption are outlined; an overview is provided in Figure 2.

In the space of biomarker discovery, the loading of data is one key challenge. As described before, biomarker discovery often necessitates extracting insights from large classical datasets. Despite progress with QRAMs, breakthroughs in this area will unlock many more applications. Related to that, quantum algorithms remain an intensely researched area. New ones are being continually discovered; for instance, a study recently claimed an exponential advantage in pathfinding for graphs.^170^ Furthermore, quantum federated learning represents another novel algorithmic area.^171^^,^^172^^,^^173^^,^^174^ The idea is to enhance federated learning models where different entities train a model while keeping their data decentralized. Given the growing importance federated learning has for healthcare and medicine, from allowing multi-institutional collaborations without patient data sharing^175^ to enabling digital health^176^ to enhancing the internet of medical things,^177^ this is a clear innovation opportunity.

In the area of biomarker validation, a critical aspect concerns benchmarks. The challenge with demonstrating value from quantum computing is that the (classical) goal posts are often not clearly defined and may shift (particularly in response to, or even inspired by, quantum algorithm advances). This makes it challenging to justify the efforts required to develop and use quantum algorithm-based tools. Connected with that, generalization is a key issue due to individual biomarker variability resulting from genetic and lifestyle factors.^178^ As a result, more granular (quantum) models will have to be developed, which might again require more (classical) data and novel quantum algorithms.

Finally, data security represents one essential area in regard to biomarker adoption. The high sensitivity of much medical data requires strict data processing standards and is not yet compatible with many quantum computing architectures that demand cloud transfer of data across the world. Similarly, clinical adoption requires a significant amount of trust to be built, which in turn necessitates explainable models and easily replicable results. This challenge, which is also closely linked to similar problems with AI adoption, is exacerbated due to the abstract and inherently probabilistic nature of quantum computing.

## Conclusion

In the long term, the hope is that discovery of better biomarkers helps pave the way toward proactive precision medicine. As opposed to lengthy reactive treatments, each individual may have a continually updated health status that is based on personalized biomarkers and indicates whenever interventions of any form could be advisable. To make this possible, biomarkers must not only be accurately connected with such a health status but must also be accessible in a cost-efficient and easy manner. This is very challenging for diseases, including multifactorial ones such as Alzheimer disease and cancer, and rare conditions for which little data exist. These are areas where quantum computing applications are particularly promising.

Clearly, there are also still discrepancies with respect to access to advanced forms of computing, such as quantum computing, across the world. While quantum computing in particular has been rapidly made more widely available, it is likely to remain relatively expensive for some time to come; today, typical costs are thousands of US dollars per QPU hour. Thus, democratization of such transformative technologies is an important issue that also needs to be considered to realize their full impact across the world.^179^

## About the authors

QuantumBasel, part of the uptownBasel innovation campus, is a center of competence for quantum computing and AI. The University of Basel is Switzerland's oldest university. Cleveland Clinic is an American nonprofit multispecialty academic medical center. DESY is a national accelerator and research center in Germany. The Cyprus Institute is a nonprofit research and educational institution with a strong scientific and technological orientation. Sandia National Laboratories is a multimission laboratory managed and operated for the US Department of Energy’s National Nuclear Security Administration. Elevance Health is an American health insurance provider. IBM is an American technology company.

## Acknowledgments

Sandia National Laboratories is a multimission laboratory managed and operated by National Technology & Engineering Solutions of Sandia, LLC, a wholly owned subsidiary of 10.13039/100019966Honeywell International Inc., for the US Department of Energy’s National Nuclear Security Administration under contract DE-NA0003525. This paper describes objective technical results and analysis. Any subjective views or opinions that might be expressed in the paper do not necessarily represent the views of the US Department of Energy or the US government.

## Declaration of interests

The authors declare no competing interests.

## References

[1] Biomarkers Definitions Working Group (2001). Biomarkers and surrogate endpoints: preferred definitions and conceptual framework. Clin. Pharmacol. Ther..

[2] Henry N.L., Hayes D.F. (2012). Cancer biomarkers. Mol. Oncol..

[3] Strimbu K., Tavel J.A. (2010). What are biomarkers?. Curr. Opin. HIV AIDS.

[4] Aronson J.K., Ferner R.E. (2017). Biomarkers—a general review. Curr. Protoc. Pharmacol..

[5] Thomas D.W., Burns J., Audette J., Carroll A., Dow-Hygelund C., Hay M. (2016). https://go.bio.org/rs/490-EHZ-999/images/Clinical%20Development%20Success%20Rates%202006-2015%20-%20BIO%2C%20Biomedtracker%2C%20Amplion%202016.pdf.

[6] Davis K.D., Aghaeepour N., Ahn A.H., Angst M.S., Borsook D., Brenton A., Burczynski M.E., Crean C., Edwards R., Gaudilliere B. (2020). Discovery and validation of biomarkers to aid the development of safe and effective pain therapeutics: challenges and opportunities. Nat. Rev. Neurol..

[7] Horowitz M., Grumbling E. (2019). Quantum Computing: Progress and Prospects.

[8] Peral-García D., Cruz-Benito J., García-Peñalvo F.J. (2024). Systematic literature review: Quantum machine learning and its applications. Computer Science Review.

[9] Jaschke D., Montangero S. (2023). Is quantum computing green? An estimate for an energy-efficiency quantum advantage. Quantum Sci. Technol..

[10] Peters E., Caldeira J., Ho A., Leichenauer S., Mohseni M., Neven H., Spentzouris P., Strain D., Perdue G.N. (2021). Machine learning of high dimensional data on a noisy quantum processor. npj Quantum Inf..

[11] Marshall S.C., Gyurik C., Dunjko V. (2023). High dimensional quantum machine learning with small quantum computers. Quantum.

[12] Felefly T., Roukoz C., Fares G., Achkar S., Yazbeck S., Meyer P., Kordahi M., Azoury F., Nasr D.N., Nasr E. (2023). An explainable MRI-radiomic quantum neural network to differentiate between large brain metastases and high-grade glioma using quantum annealing for feature selection. J. Digit. Imag..

[13] Mauranyapin N.P., Terrasson A., Bowen W.P. (2022). Quantum biotechnology. Adv. Quantum Technol..

[14] Flöther F.F., Griffin P.F. (2023). How can quantum technologies be applied in healthcare, medicine and the life sciences?. Res. dir. Quantum technol..

[15] Shams M., Choudhari J., Reyes K., Prentzas S., Gapizov A., Shehryar A., Affaf M., Grezenko H., Gasim R.W., Mohsin S.N. (2023). The Quantum-Medical Nexus: Understanding the Impact of Quantum Technologies on Healthcare. Cureus.

[16] Schuld M., Petruccione F. (2018).

[17] Havlíček V., Córcoles A.D., Temme K., Harrow A.W., Kandala A., Chow J.M., Gambetta J.M. (2019). Supervised learning with quantum-enhanced feature spaces. Nature.

[18] Nakaji K., Uno S., Suzuki Y., Raymond R., Onodera T., Tanaka T., Tezuka H., Mitsuda N., Yamamoto N. (2022). Approximate amplitude encoding in shallow parameterized quantum circuits and its application to financial market indicators. Phys. Rev. Res..

[19] Cerezo M., Arrasmith A., Babbush R., Benjamin S.C., Endo S., Fujii K., McClean J.R., Mitarai K., Yuan X., Cincio L., Coles P.J. (2021). Variational quantum algorithms. Nat. Rev. Phys..

[20] Cai Z., Babbush R., Benjamin S.C., Endo S., Huggins W.J., Li Y., McClean J.R., O’Brien T.E. (2023). Quantum error mitigation. Rev. Mod. Phys..

[21] Bayerstadler A., Becquin G., Binder J., Botter T., Ehm H., Ehmer T., Erdmann M., Gaus N., Harbach P., Hess M. (2021). Industry quantum computing applications. EPJ Quantum Technol..

[22] Bowles J., Ahmed S., Schuld M. (2024). Better than classical? The subtle art of benchmarking quantum machine learning models. arXiv.

[23] Schuld M., Bocharov A., Svore K.M., Wiebe N. (2020). Circuit-centric quantum classifiers. Phys. Rev. A.

[24] Pérez-Salinas A., Cervera-Lierta A., Gil-Fuster E., Latorre J.I. (2020). Data re-uploading for a universal quantum classifier. Quantum.

[25] Huang H.Y., Broughton M., Mohseni M., Babbush R., Boixo S., Neven H., McClean J.R. (2021). Power of data in quantum machine learning. Nat. Commun..

[26] Caro M.C., Huang H.Y., Cerezo M., Sharma K., Sornborger A., Cincio L., Coles P.J. (2022). Generalization in quantum machine learning from few training data. Nat. Commun..

[27] Cerezo M., Larocca M., García-Martín D., Diaz N.L., Braccia P., Fontana E., Rudolph M.S., Bermejo P., Ijaz A., Thanaslip S. (2023). Does provable absence of barren plateaus imply classical simulability? or, why we need to rethink variational quantum computing. arXiv.

[28] Larocca M., Thanasilp S., Wang S., Sharma K., Biamonte J., Coles P.J., Cincio L., McClean J.R., Holmes Z., Cerezo M. (2024). A review of barren plateaus in variational quantum computing. arXiv.

[29] Lloyd S., Mohseni M., Rebentrost P. (2014). Quantum principal component analysis. Nat. Phys..

[30] Gordon M.H., Cerezo M., Cincio L., Coles P.J. (2022). Covariance Matrix Preparation for Quantum Principal Component Analysis. PRX Quantum.

[31] Yu K., Lin S., Guo G.D. (2023). Quantum dimensionality reduction by linear discriminant analysis. Phys. Stat. Mech. Appl..

[32] Kerenidis I., Luongo A. (2020). Classification of the MNIST data set with quantum slow feature analysis. Phys. Rev. A.

[33] Feng W., Guo G., Lin S., Xu Y. (2024). Quantum Isomap algorithm for manifold learning. Phys. Rev. Appl..

[34] Cong I., Choi S., Lukin M.D. (2019). Quantum convolutional neural networks. Nat. Phys..

[35] Ray A., Madan D., Patil S., Pati P., Rapsomaniki M., Kohlakala A., Dlamini T.R., Muller S.J., Rhrissorrakrai K., Utro F., Parida L. (2024). 2024 IEEE International Conference on Quantum Computing and Engineering (QCE).

[36] Wiebe N., Braun D., Lloyd S. (2012). Quantum Algorithm for Data Fitting. Phys. Rev. Lett..

[37] Wang G. (2017). Quantum algorithm for linear regression. Phys. Rev. A.

[38] Zhao Z., Fitzsimons J.K., Fitzsimons J.F. (2019). Quantum-assisted Gaussian process regression. Phys. Rev. A.

[39] Date P., Potok T. (2021). Adiabatic quantum linear regression. Sci. Rep..

[40] Khan S.U., Awan A.J., Vall-Llosera G. (2019). K-means clustering on noisy intermediate scale quantum computers. arXiv.

[41] Arthur D., Date P. (2021). Balanced k-means clustering on an adiabatic quantum computer. Quant. Inf. Process..

[42] Kerenidis I., Landman J. (2021). Quantum spectral clustering. Phys. Rev. A.

[43] Tian J., Sun X., Du Y., Zhao S., Liu Q., Zhang K., Yi W., Huang W., Wang C., Wu X. (2023). Recent advances for quantum neural networks in generative learning. IEEE Trans. Pattern Anal. Mach. Intell..

[44] Amin M.H., Andriyash E., Rolfe J., Kulchytskyy B., Melko R. (2018). Quantum Boltzmann Machine. Phys. Rev. X.

[45] Tüysüz C., Demidik M., Coopmans L., Rinaldi E., Croft V., Haddad Y., Rosenkranz M., Jansen K. (2024). Learning to generate high-dimensional distributions with low-dimensional quantum Boltzmann machines. arXiv.

[46] Baglio J. (2024). Data augmentation experiments with style-based quantum generative adversarial networks on trapped-ion and superconducting-qubit technologies. arXiv.

[47] Suzuki Y., Gao Q., Pradel K.C., Yasuoka K., Yamamoto N. (2022). Natural quantum reservoir computing for temporal information processing. Sci. Rep..

[48] Di Sipio R., Huang J.H., Chen S.Y.C., Mangini S., Worring M. (2022). ICASSP 2022-2022 IEEE International Conference on Acoustics, Speech and Signal Processing (ICASSP).

[49] Choi J., Kim J. (2019). 2019 international conference on information and communication technology convergence (ICTC).

[50] Flori A., Oulhadj H., Siarry P. (2022). QUAntum Particle Swarm Optimization: An auto-adaptive PSO for local and global optimization. Comput. Optim. Appl..

[51] Tilly J., Chen H., Cao S., Picozzi D., Setia K., Li Y., Grant E., Wossnig L., Rungger I., Booth G.H., Tennyson J. (2022). The variational quantum eigensolver: a review of methods and best practices. Phys. Rep..

[52] Guarasci R., De Pietro G., Esposito M. (2022). Quantum natural language processing: Challenges and opportunities. Appl. Sci..

[53] Emani P.S., Warrell J., Anticevic A., Bekiranov S., Gandal M., McConnell M.J., Sapiro G., Aspuru-Guzik A., Baker J.T., Bastiani M. (2021). Quantum computing at the frontiers of biological sciences. Nat. Methods.

[54] Basu S., Born J., Bose A., Capponi S., Chalkia D., Chan T.A., Doga H., Flother F.F., Getz G., Goldsmith M. (2023). Towards quantum-enabled cell-centric therapeutics. arXiv.

[55] Doga H., Bose A., Sahin M.E., Bettencourt-Silva J., Pham A., Kim E. (2024). How can quantum computing be applied in clinical trial design and optimization?. Trends Pharmacol. Sci..

[56] Gupta R.S., Wood C.E., Engstrom T., Pole J.D., Shrapnel S. (2024). Quantum Machine Learning for Digital Health? A Systematic Review. arXiv.

[57] Flöther F.F. (2023). The state of quantum computing applications in health and medicine. Research Directions: Quantum Technologies.

[58] Sorkhabi L.B., Gharehchopogh F.S., Shahamfar J. (2020). A systematic approach for pre-processing electronic health records for mining: Case study of heart disease. Int. J. Data Min. Bioinf..

[59] Torres-Martos Á., Bustos-Aibar M., Ramírez-Mena A., Cámara-Sánchez S., Anguita-Ruiz A., Alcalá R., Aguilera C.M., Alcalá-Fdez J. (2023). Omics data preprocessing for machine learning: A case study in childhood obesity. Genesis.

[60] Schuld M., Killoran N. (2019). Quantum machine learning in feature hilbert spaces. Phys. Rev. Lett..

[61] Shilo S., Rossman H., Segal E. (2020). Axes of a revolution: challenges and promises of big data in healthcare. Nat. Med..

[62] Maniscalco S., Borrelli E.M., Cavalcanti D., Foti C., Glos A., Goldsmith M., Knecht S., Korhonen K., Malmi J., Nykänen A. (2022). Quantum network medicine: rethinking medicine with network science and quantum algorithms. arXiv.

[63] Sagingalieva A., Kordzanganeh M., Kenbayev N., Kosichkina D., Tomashuk T., Melnikov A. (2023). Hybrid quantum neural network for drug response prediction. Cancer.

[64] Prins B.P., Kuchenbaecker K.B., Bao Y., Smart M., Zabaneh D., Fatemifar G., Luan J., Wareham N.J., Scott R.A., Perry J.R.B. (2017). Genome-wide analysis of health-related biomarkers in the UK Household Longitudinal Study reveals novel associations. Sci. Rep..

[65] Bubeck S., Sellke M. (2023). A universal law of robustness via isoperimetry. J. ACM.

[66] Kaplan J., McCandlish S., Henighan T., Brown T.B., Chess B., Child R., Gray S., Radford A., Wu J., Amodei D. (2020). Scaling laws for neural language models. arXiv.

[67] Hoffmann J., Borgeaud S., Mensch A., Buchatskaya E., Cai T., Rutherford E., de Las Casas D., Hendricks L.A., Welbl J., Clark J. (2022). Training compute-optimal large language models. arXiv.

[68] Tang E. (2021). Quantum Principal Component Analysis Only Achieves an Exponential Speedup Because of Its State Preparation Assumptions. Phys. Rev. Lett..

[69] Phalak K., Chatterjee A., Ghosh S. (2023). Quantum random access memory for dummies. Sensors.

[70] Harrow A.W. (2020). Small quantum computers and large classical data sets. arXiv.

[71] Yogendran B., Charlton D., Beddig M., Kolotouros I., Wallden P. (2024). Big data applications on small quantum computers. arXiv.

[72] Chen M., Tan X., Padman R. (2020). Social determinants of health in electronic health records and their impact on analysis and risk prediction: a systematic review. J. Am. Med. Inf. Assoc..

[73] Ravizza S., Huschto T., Adamov A., Böhm L., Büsser A., Flöther F.F., Hinzmann R., König H., McAhren S.M., Robertson D.H. (2019). Predicting the early risk of chronic kidney disease in patients with diabetes using real-world data. Nat. Med..

[74] Li Y., Rao S., Solares J.R.A., Hassaine A., Ramakrishnan R., Canoy D., Zhu Y., Rahimi K., Salimi-Khorshidi G. (2020). BEHRT: transformer for electronic health records. Sci. Rep..

[75] Adamson B., Waskom M., Blarre A., Kelly J., Krismer K., Nemeth S., Gippetti J., Ritten J., Harrison K., Ho G. (2023). Approach to machine learning for extraction of real-world data variables from electronic health records. Front. Pharmacol..

[76] Singhal P., Tan A.L.M., Drivas T., Johnson K., Ritchie M., Beaulieu-Jones B. (2023). Opportunities and challenges for biomarker discovery using electronic health record data. Trends Mol. Med..

[77] Mosley J.D., Feng Q., Wells Q.S., Van Driest S.L., Shaffer C.M., Edwards T.L., Bastarache L., Wei W.Q., Davis L.K., McCarty C.A. (2018). A study paradigm integrating prospective epidemiologic cohorts and electronic health records to identify disease biomarkers. Nat. Commun..

[78] Mohsen F., Ali H., El Hajj N., Shah Z. (2022). Artificial intelligence-based methods for fusion of electronic health records and imaging data. Sci. Rep..

[79] Steyaert S., Pizurica M., Nagaraj D., Khandelwal P., Hernandez-Boussard T., Gentles A.J., Gevaert O. (2023). Multimodal data fusion for cancer biomarker discovery with deep learning. Nat. Mach. Intell..

[80] Assale M., Dui L.G., Cina A., Seveso A., Cabitza F. (2019). The revival of the notes field: leveraging the unstructured content in electronic health records. Front. Med..

[81] Tam V., Patel N., Turcotte M., Bossé Y., Paré G., Meyre D. (2019). Benefits and limitations of genome-wide association studies. Nat. Rev. Genet..

[82] Uffelmann E., Huang Q.Q., Munung N.S., de Vries J., Okada Y., Martin A.R., Martin H.C., Lappalainen T., Posthuma D. (2021). Genome-wide association studies. Nat. Rev. Methods Primers.

[83] Donoho D.L. (2000). High-dimensional data analysis: The curses and blessings of dimensionality. AMS math challenges lecture..

[84] Pudjihartono N., Fadason T., Kempa-Liehr A.W., O’Sullivan J.M. (2022). A review of feature selection methods for machine learning-based disease risk prediction. Front. Bioinform..

[85] Nguyen P.N. (2024). Biomarker discovery with quantum neural networks: a case-study in CTLA4-activation pathways. BMC Bioinf..

[86] Saggi M.K., Bhatia A.S., Isaiah M., Gowher H., Kais S. (2024). Multi-Omic and quantum machine learning integration for lung subtypes classification. arXiv.

[87] Albert F.W., Kruglyak L. (2015). The role of regulatory variation in complex traits and disease. Nat. Rev. Genet..

[88] Nałęcz-Charkiewicz K., Charkiewicz K., Nowak R.M. (2024). Quantum computing in bioinformatics: a systematic review mapping. Brief. Bioinform.

[89] Price A.L., Patterson N.J., Plenge R.M., Weinblatt M.E., Shadick N.A., Reich D. (2006). Principal components analysis corrects for stratification in genome-wide association studies. Nat. Genet..

[90] Li Z., Chai Z., Guo Y., Ji W., Wang M., Shi F., Wang Y., Lloyd S., Du J. (2021). Resonant quantum principal component analysis. Sci. Adv..

[91] Uffelmann E., Posthuma D. (2021). Emerging methods and resources for biological interrogation of neuropsychiatric polygenic signal. Biol. Psychiatry..

[92] Holland D., Frei O., Desikan R., Fan C.C., Shadrin A.A., Smeland O.B., Sundar V.S., Thompson P., Andreassen O.A., Dale A.M. (2020). Beyond SNP heritability: Polygenicity and discoverability of phenotypes estimated with a univariate Gaussian mixture model. PLoS Genet..

[93] Slatkin M. (2008). Linkage disequilibrium—understanding the evolutionary past and mapping the medical future. Nat. Rev. Genet..

[94] Navascués M., Wolfe E. (2020). The Inflation Technique Completely Solves the Causal Compatibility Problem. J. Causal Inference.

[95] Chiribella G., Ebler D. (2019). Quantum speedup in the identification of cause–effect relations. Nat. Commun..

[96] Nica A.C., Dermitzakis E.T. (2013). Expression quantitative trait loci: present and future. Philos. Trans. R. Soc. Lond. B Biol. Sci..

[97] Khalsan M., Machado L.R., Al-Shamery E.S., Ajit S., Anthony K., Mu M., Agyeman M.O. (2022). A survey of machine learning approaches applied to gene expression analysis for cancer prediction. IEEE Access.

[98] Chen Z., Chen S., Qiang X. (2022). Identification of Biomarker in Brain-specific Gene Regulatory Network Using Structural Controllability Analysis. Front. Bioinform..

[99] Zhang Z., Sun C., Liu Z.P. (2022). Discovering biomarkers of hepatocellular carcinoma from single-cell RNA sequencing data by cooperative games on gene regulatory network. Journal of Computational Science.

[100] Roman-Vicharra C., Cai J.J. (2023). Quantum gene regulatory networks. npj Quantum Inf..

[101] Osorio D., Yu X., Yu P., Serpedin E., Cai J.J. (2019). Single-cell RNA sequencing of a European and an African lymphoblastoid cell line. Sci. Data.

[102] SoRelle E.D., Dai J., Bonglack E.N., Heckenberg E.M., Zhou J.Y., Giamberardino S.N., Bailey J.A., Gregory S.G., Chan C., Luftig M.A. (2021). Single-cell RNA-seq reveals transcriptomic heterogeneity mediated by host–pathogen dynamics in lymphoblastoid cell lines. Elife.

[103] Al-Janabee O., Al-Sarray B. (2023). Review of clustering for gene expression data. AIP Conf. Proc..

[104] Rodriguez M.Z., Comin C.H., Casanova D., Bruno O.M., Amancio D.R., Costa L.F., Rodrigues F.A. (2019). Clustering algorithms: A comparative approach. PLoS One.

[105] Wang D., Tan D., Liu L. (2018). Particle swarm optimization algorithm: an overview. Soft Comput..

[106] Sun J., Feng B., Xu W. (2004). Particle swarm optimization with particles having quantum behavior. Proceedings of the 2004 Congress on Evolutionary Computation (IEEE Cat. No.04TH8753).

[107] Clerc M., Kennedy J. (2002). The particle swarm - explosion, stability, and convergence in a multidimensional complex space. IEEE Trans. Evol. Comput..

[108] van den Bergh F. (2001). An Analysis of Particle Swarm Optimizers.

[109] Chen W., Sun J., Ding Y., Fang W., Xu W., Nguyen N.T., Borzemski L., Grzech A., Ali M. (2008). New Frontiers in Applied Artificial Intelligence.

[110] Sun J., Chen W., Fang W., Wun X., Xu W. (2012). Gene expression data analysis with the clustering method based on an improved quantum-behaved Particle Swarm Optimization. Eng. Appl. Artif. Intell..

[111] Dabba A., Tari A., Meftali S. (2020). Hybridization of Moth flame optimization algorithm and quantum computing for gene selection in microarray data. J. Ambient Intell. Hum. Comput..

[112] Fallahi S., Taghadosi M. (2022). Quantum-behaved particle swarm optimization based on solitons. Sci. Rep..

[113] Elaraby A. (2022). Quantum medical images processing foundations and applications. IET Quantum Communication.

[114] Wei L., Liu H., Xu J., Shi L., Shan Z., Zhao B., Gao Y. (2023). Quantum machine learning in medical image analysis: A survey. Neurocomputing.

[115] Zack M.M., Kobau R. (2017). National and state estimates of the numbers of adults and children with active epilepsy—United States, 2015. MMWR Morb. Mortal. Wkly. Rep..

[116] Anuradha R., Vandana C., Singh S.V., Singh N., Emad R., Nitin V. (2024). 2024 International Conference on Communication, Computer Sciences and Engineering (IC3SE).

[117] Yang L., Vetter C., Qiu F. (2020). Volume rendering from three-dimensional medical data using quantum computing. US patent US20200242816A1.

[118] Cascarano A., Mur-Petit J., Hernández-González J., Camacho M., de Toro Eadie N., Gkontra P., Chadeau-Hyam M., Vitrià J., Lekadir K. (2023). Machine and deep learning for longitudinal biomedical data: a review of methods and applications. Artif. Intell. Rev..

[119] Snyder-Mackler N., Burger J.R., Gaydosh L., Belsky D.W., Noppert G.A., Campos F.A., Bartolomucci A., Yang Y.C., Aiello A.E., O'Rand A. (2020). Social determinants of health and survival in humans and other animals. Science.

[120] Mujal P., Martínez-Peña R., Giorgi G.L., Soriano M.C., Zambrini R. (2023). Time-series quantum reservoir computing with weak and projective measurements. npj Quantum Inf..

[121] Daskin A. (2022). A walk through of time series analysis on quantum computers. arXiv.

[122] Benjamin M.A., Rigby R.A., Stasinopoulos D.M. (2003). Generalized autoregressive moving average models. J. Am. Stat. Assoc..

[123] Van Houdt G., Mosquera C., Nápoles G. (2020). A review on the long short-term memory model. Artif. Intell. Rev..

[124] Mujal P., Martínez-Peña R., Nokkala J., García-Beni J., Giorgi G.L., Soriano M.C. (2021). Opportunities in quantum reservoir computing and extreme learning machines. Advanced Quantum Technologies.

[125] Takaki Y., Mitarai K., Negoro M., Fujii K., Kitagawa M. (2021). Learning temporal data with a variational quantum recurrent neural network. Phys. Rev. A.

[126] Lukoševičius M. (2012). Neural Networks: Tricks of the Trade.

[127] Fernando C., Sojakka S. (2003). European conference on artificial life.

[128] Tanaka G., Yamane T., Héroux J.B., Nakane R., Kanazawa N., Takeda S., Numata H., Nakano D., Hirose A. (2019). Recent advances in physical reservoir computing: A review. Neural Netw..

[129] Komariah K.S., Sin B.K. (2019). 2019 Eleventh International Conference on Ubiquitous and Future Networks (ICUFN).

[130] Dinh-Le C., Chuang R., Chokshi S., Mann D. (2019). Wearable health technology and electronic health record integration: scoping review and future directions. JMIR Mhealth Uhealth.

[131] Sempionatto J.R., Lasalde-Ramírez J.A., Mahato K., Wang J., Gao W. (2022). Wearable chemical sensors for biomarker discovery in the omics era. Nat. Rev. Chem.

[132] Kourtis L.C., Regele O.B., Wright J.M., Jones G.B. (2019). Digital biomarkers for Alzheimer’s disease: the mobile/wearable devices opportunity. npj Digit. Med..

[133] Aslam N., Zhou H., Urbach E.K., Turner M.J., Walsworth R.L., Lukin M.D., Park H. (2023). Quantum sensors for biomedical applications. Nat. Rev. Phys..

[134] Hershberger C.E., Louis S., Busch R.M., Vegh D., Najm I., Bazeley P., Eng C., Jehi L., Rotroff D.M. (2023). Molecular subtypes of epilepsy associated with post-surgical seizure recurrence. Brain Commun..

[135] Mihajlović K., Ceddia G., Malod-Dognin N., Novak G., Kyriakis D., Skupin A., Pržulj N. (2024). Multi-omics integration of scRNA-seq time series data predicts new intervention points for Parkinson’s disease. Sci. Rep..

[136] Parry E.M., Leshchiner I., Guièze R., Johnson C., Tausch E., Parikh S.A., Lemvigh C., Broséus J., Hergalant S., Messer C. (2023). Evolutionary history of transformation from chronic lymphocytic leukemia to Richter syndrome. Nat. Med..

[137] Parsons H.A., Messer C., Santos K., Danysh B.P., Hughes M.E., Patel A., Jacobs R.A., Slowik K., Hess J., Stewart C. (2023). Abstract 3874: Genomic mechanisms of resistance to tyrosine kinase inhibitors (TKIs) in HER2+ metastatic breast cancer (HER2+ MBC). Cancer Res..

[138] Naeem A., Li L., Utro F., Cha J., Tsuji J., Fernandes S.M., Azevedo R.S., Morelli F., Wang Z., Shupe S.J. (2023). Understanding Resistance Mechanisms and Growth Kinetics of CLL Treated with Covalent and Non-Covalent BTK Inhibitors. Blood.

[139] Burr R., Leshchiner I., Costantino C.L., Blohmer M., Sundaresan T., Cha J., Seeger K., Guay S., Danysh B.P., Gore I. (2024). Developmental mosaicism underlying *EGFR*-mutant lung cancer presenting with multiple primary tumors. Nat. Cancer.

[140] Khan K.H., Cunningham D., Werner B., Vlachogiannis G., Spiteri I., Heide T., Mateos J.F., Vatsiou A., Lampis A., Damavandi M.D. (2018). Longitudinal Liquid Biopsy and Mathematical Modeling of Clonal Evolution Forecast Time to Treatment Failure in the PROSPECT-C Phase II Colorectal Cancer Clinical Trial. Cancer Discov..

[141] Mathios D., Johansen J.S., Cristiano S., Medina J.E., Phallen J., Larsen K.R., Bruhm D.C., Niknafs N., Ferreira L., Adleff V. (2021). Detection and characterization of lung cancer using cell-free DNA fragmentomes. Nat. Commun..

[142] Rolfo C., Russo A. (2023). Brave new world of cfDNA-omics for early cancer detection. J. Immunother. Cancer.

[143] Shi M., Mollah S. (2021). NeTOIF: A Network-based Approach for Time-Series Omics Data Imputation and Forecasting. bioRxiv.

[144] Ruiz-Perez D., Lugo-Martinez J., Bourguignon N., Mathee K., Lerner B., Bar-Joseph Z., Narasimhan G. (2021). Dynamic bayesian networks for integrating multi-omics time series microbiome data. mSystems.

[145] Hou K., Wu Z.X., Chen X.Y., Wang J.Q., Zhang D., Xiao C., Zhu D., Koya J.B., Wei L., Li J., Chen Z.S. (2022). Microbiota in health and diseases. Signal Transduct. Target. Ther..

[146] Buytaers F.E., Berger N., Van der Heyden J., Roosens N.H.C., De Keersmaecker S.C.J. (2024). The potential of including the microbiome as biomarker in population-based health studies: methods and benefits. Front. Public Health.

[147] Gyurik C., Schmidhuber A., King R., Dunjko V., Hayakawa R. (2024). Quantum computing and persistence in topological data analysis. arXiv.

[148] He K., Zhang X., Ren S., Sun J. (2016). Proceedings of the IEEE conference on computer vision and pattern recognition.

[149] Du Y., Hsieh M.H., Liu T., Tao D., Liu N. (2021). Quantum noise protects quantum classifiers against adversaries. Phys. Rev. Res..

[150] Resch S., Karpuzcu U.R. (2021). Benchmarking quantum computers and the impact of quantum noise. ACM Comput. Surv..

[151] Kiwit F.J., Wolf M.A., Marso M., Ross P., Lorenz J.M., Riofrío C.A., Luckow A. (2024). Benchmarking quantum generative learning: A study on scalability and noise resilience using QUARK. Künstl. Intell..

[152] Monzani F., Ricci E., Nigro L., Prati E. (2024). Leveraging non-unital noise for gate-based quantum reservoir computing. arXiv.

[153] Sivak V.V., Eickbusch A., Royer B., Singh S., Tsioutsios I., Ganjam S., Miano A., Brock B.L., Ding A.Z., Frunzio L. (2023). Real-time quantum error correction beyond break-even. Nature.

[154] Karimi D., Dou H., Warfield S.K., Gholipour A. (2020). Deep learning with noisy labels: Exploring techniques and remedies in medical image analysis. Med. Image Anal..

[155] Algan G., Ulusoy I. (2021). Image classification with deep learning in the presence of noisy labels: A survey. Knowl. Base Syst..

[156] Song H., Kim M., Park D., Shin Y., Lee J.G. (2023). Learning from noisy labels with deep neural networks: A survey. IEEE Transact. Neural Networks Learn. Syst..

[157] Liang W., Tadesse G.A., Ho D., Fei-Fei L., Zaharia M., Zhang C., Zou J. (2022). Advances, challenges and opportunities in creating data for trustworthy AI. Nat. Mach. Intell..

[158] Khatun A., Aydeniz K.Y., Weinstein Y.S., Usman M. (2024). Quantum Generative Learning for High-Resolution Medical Image Generation. arXiv.

[159] Guan J., Fang W., Ying M. (2022). International Conference on Computer Aided Verification.

[160] Otgonbaatar S., Schwarz G., Datcu M., Kranzlmüller D. (2023). Quantum transfer learning for real-world, small, and high-dimensional remotely sensed datasets. IEEE J. Sel. Top. Appl. Earth Obs. Remote Sens..

[161] Maheshwari D., Ullah U., Marulanda P.A.O., Jurado A.G.O., Gonzalez I.D., Merodio J.M.O., Garcia-Zapirain B. (2023). Quantum machine learning applied to electronic healthcare records for ischemic heart disease classification. Hum-Cent Comput Inf Sci.

[162] Krunic Z., Flöther F.F., Seegan G., Earnest-Noble N., Omar S. (2022). Quantum kernels for real-world predictions based on electronic health records. IEEE Trans. Quantum Eng..

[163] Hibat-Allah M., Mauri M., Carrasquilla J., Perdomo-Ortiz A. (2024). A framework for demonstrating practical quantum advantage: comparing quantum against classical generative models. Commun. Phys..

[164] Kazdaghli S., Kerenidis I., Kieckbusch J., Teare P. (2024). Improved clinical data imputation via classical and quantum determinantal point processes. Elife.

[165] Wang L., Liu Y., Meng F., Luan T., Liu W., Zhang Z., Yu X. (2024). A quantum synthetic aperture radar image denoising algorithm based on grayscale morphology. iScience.

[166] Zhou Y., Wong Y.K., Liang Y.S. (2024). Sixteenth International Conference on Digital Image Processing (ICDIP 2024).

[167] Haque M.E., Paul M., Ulhaq A., Debnath T. (2023). Advanced quantum image representation and compression using a DCT-EFRQI approach. Sci. Rep..

[168] Deb S.K., Pan W.D. (2024). Quantum Image Compression: Fundamentals, Algorithms, and Advances. Computers.

[169] Ben Yedder H., Cardoen B., Hamarneh G. (2021). Deep learning for biomedical image reconstruction: A survey. Artif. Intell. Rev..

[170] Li J., Tong Y. (2024). Exponential Quantum Advantage for Pathfinding in Regular Sunflower Graphs. arXiv.

[171] Li W., Lu S., Deng D.L. (2021). Quantum federated learning through blind quantum computing. Sci. China Phys. Mech. Astron..

[172] Chehimi M., Saad W. (2022). ICASSP 2022-2022 IEEE International Conference on Acoustics, Speech and Signal Processing (ICASSP).

[173] Huang R., Tan X., Xu Q. (2022). Quantum federated learning with decentralized data. IEEE J. Sel. Top. Quant. Electron..

[174] Bhatia A.S., Kais S., Alam M.A. (2023). Federated quanvolutional neural network: a new paradigm for collaborative quantum learning. Quantum Sci. Technol..

[175] Sheller M.J., Edwards B., Reina G.A., Martin J., Pati S., Kotrotsou A., Milchenko M., Xu W., Marcus D., Colen R.R., Bakas S. (2020). Federated learning in medicine: facilitating multi-institutional collaborations without sharing patient data. Sci. Rep..

[176] Rieke N., Hancox J., Li W., Milletarì F., Roth H.R., Albarqouni S., Bakas S., Galtier M.N., Landman B.A., Maier-Hein K. (2020). The future of digital health with federated learning. npj Digit. Med..

[177] Prasad V.K., Bhattacharya P., Maru D., Tanwar S., Verma A., Singh A., Tiwari A.K., Sharma R., Alkhayyat A., Țurcanu F.E., Raboaca M.S. (2022). Federated learning for the internet-of-medical-things: A survey. Mathematics.

[178] Enroth S., Johansson Å., Enroth S.B., Gyllensten U. (2014). Strong effects of genetic and lifestyle factors on biomarker variation and use of personalized cutoffs. Nat. Commun..

[179] Seskir Z.C., Umbrello S., Coenen C., Vermaas P.E. (2023). Democratization of quantum technologies. Quantum Sci. Technol..

